# Clinicopathological correlations in 38 cases of gastroenteropancreatic high-grade neuroendocrine neoplasms

**DOI:** 10.3389/fonc.2024.1399079

**Published:** 2024-10-17

**Authors:** Na Li, Yanping Hu, Linguo Wu, Jianduo An

**Affiliations:** Department of Pathology, Beijing Luhe Hospital, Capital Medical University, Beijing, China

**Keywords:** gastroenteropancreatic high-grade neuroendocrine neoplasms, NET G3, Ki-67, *BRAF V600E*, clinicopathological features, prognostic analysis

## Abstract

**Objective:**

Diagnosis and treatment of gastroenteropancreatic high-grade neuroendocrine neoplasms (GEP-HG-NENs), particularly G3 well-differentiated neuroendocrine tumours (NETs) and poorly differentiated neuroendocrine carcinomas (NECs) relies on histopathological morphology, immunohistochemistry, and molecular biological markers, which are lacking especially in cases with ambiguous histomorphology. In this study to contribute to the development of more targeted treatment strategies, we examined various immunohistochemical and molecular biological markers and their association with clinicopathological features in GEP-HG-NENs.

**Methods:**

We included 38 patients with GEP-HG-NENs in this study, with their retrospective follow-up data. The expression of tumour protein p53 (TP53), RB transcriptional corepressor 1 (RB1), somatostatin receptor 2 (SSTR2), clusterin (CLU), and marker of proliferation Ki-67 (MKI67) was immunohistochemically analysed. KRAS proto-oncogene, GTPase (*KRAS*) and B-Raf proto-oncogene, serine/threonine kinase (*BRAF*) *V600E* expression was evaluated using quantitative real-time polymerase chain reaction (qRT-PCR). The relationships between immunohistochemical and molecular biological markers and clinicopathological characteristics were examined using a Cox risk regression model, receiver operating characteristic (ROC) curve, and Kaplan–Meier survival analyses.

**Results:**

SSTR2, RB, TP53, and CLU expression differed between NET G3 and NECs, with variations among the NET G3 and small- and large-cell NEC (SCNEC and LCNEC, respectively) groups (*p <* 0.05). The median MKI67 proliferative index was approximately 40% and 70% in G3 NETs and NECs, respectively. The NET G3 group exhibited a median survival of 25 months, indicating a relatively better prognosis than that of the NECs group (median survival, 11 months). Both Kaplan–Meier survival analysis and the Cox risk regression model indicated a statistical correlation among treatment methods, CLU expression, and prognosis (*p <* 0.05). The *BRAF V600E* mutation rate was 32.4% in G3 NETs and SCNEC, demonstrating a significant difference between both types (*p =* 0.0086). Furthermore, ROC curve analysis highlighted the diagnostic significance of the positive expression of the immunohistochemical markers CLU, SSTR2, and RB in identifying NET G3.

**Conclusion:**

To guide more suitable treatment strategies, it is essential to develop and apply valuable and more targeted immunohistochemical and molecular pathological markers for a comprehensive analysis.

## 1 Introduction

Gastroenteropancreatic neuroendocrine neoplasms (GEP-NENs) encompass various tumours originating from neuroendocrine cells within mucosal glands of the gastroenteropancreatic system. A new category called “highly proliferative neuroendocrine tumours” has been introduced for cases showing good morphological differentiation but a high MKI67 proliferation index, as supported by previous clinical and pathological findings (1). The 2019 World Health Organization (WHO) Classification of Tumours of the Digestive System categorises these tumours into well-differentiated neuroendocrine tumours (NETs) and poorly differentiated neuroendocrine carcinomas (NECs) according to their distinct morphology (1).

The NETs category has three subcategories: low-, intermediate-, and high-grade (NET G1, G2, and G3, respectively). The fifth edition of the WHO classification further refined the classification of NENs based on their proliferative activity into high-grade (HG)-NENs, which included NET G3 and NECs (>20 mitoses/2 mm² or MKI67 proliferation index >20%). Recent research suggests that relying solely on MKI67 proliferation index >20% may not provide a comprehensive prognostic assessment of NET G3 or NECs. A previous study reported significant differences in biological behaviour, treatment options, and survival rates between HG-NENs with an MKI67 proliferation index of 20–55% compared to those with an MKI67 index of ≥55% (2).

Although the definition of HG-NENs is clear, G3 NETs and NECs have distinct pathomorphological and molecular characteristics, and differ in epidemiology, treatment, and prognosis (3). This is indicative of the pathological heterogeneity of HG-NENs and differences in their biological behaviour (4). In addition, in the initial stages of clinical discovery, certain HG-NENs cases are associated with metastasis or infiltration; however, deep infiltration and liver and lung metastases may have been present at the time of diagnosis. Additionally, the degeneration in the biological behaviour of NECs is substantially worse than that of NET G3 (5). Distinguishing between NET G3 and NECs is often challenging because of their similar pathological appearances, which further contributes to difficulties in their clinical diagnosis and treatment. Furthermore, multivariate analysis of the disease clinicopathology has associated advanced age, specific type of HG-NENs, higher tumour proliferative activity, and the presence of distant metastases with poor clinical prognosis (6).

Immunohistochemistry can be used to detect specific polypeptide hormones and bioactive amines secreted by tumour cells, which aids in the diagnosis and grading of HG-NENs. The fifth edition of the WHO grading system for GEP-NENs defines HG-NENs as having an MKI67 proliferation index <20%. However, distinguishing between G3 NETs and NECs requires additional evaluation of the level of cell differentiation. Some studies suggest an MKI67 proliferation index of 55% as the threshold, indicating differences in behaviour, treatment, and prognosis among NET G3 subgroups (2). Antibodies against somatostatin receptor 2A (SSTR2A), TP53, RB, cyclin dependent kinase inhibitor 2A (CDKN2A [P16]), and BCL2 apoptosis regulator (BCL2) are commonly used to assess the genetic status of sporadic pancreatic NETs and NECs, with a higher prevalence of TP53 mutations and RB protein inactivation in NECs (7–9). SSTR2A expression is higher in well-differentiated NETs than it is in poorly differentiated NECs. Clusterin (CLU) expression is notably high in LG-NENs (10), but further research is needed to determine whether these indicators show distinct patterns in gastroenteropancreatic NETs.

Recent advancements in molecular pathology have provided further insight into the distinct molecular pathogenesis of HG-NENs. Genome-wide studies have revealed that pancreatic NETs exhibit molecular alterations in several key pathways, including DNA damage repair, chromatin remodelling, telomere alterations, and the phosphatidylinositol-4,5-bisphosphate 3 kinase (PI3K)/mechanistic target of rapamycin kinase (MTOR) signalling pathway (11, 12). Similarly, mutated genes such as *TP53*, phosphatase and tensin homolog (*PTEN*), cyclin dependent kinase inhibitor 2A (*CDKN2A*), F-box and WD repeat domain containing 7 (*FBXW7*), and AKT serine/threonine kinase 1 (*AKT1*) have been detected in rectal NETs (13, 14).

In contrast, NECs follow a different molecular pathway characterised by mutations in genes such as *TP53*, *RB1*, KRAS proto-oncogene, GTPase (*KRAS*), B-Raf proto-oncogene, serine/threonine kinase (*BRAF*), APC regulator of WNT signalling pathway (*APC*). *TP53* mutations are rare in NETs, and *RB1* gene mutation is absent in NETs (15). Some studies suggest that *TP53* mutations are limited to certain G3 NETs (16). Most research in this field relies on high-throughput sequencing technology, which requires high-quality specimens from tumour core regions for accurate sequencing. Obtaining fresh tumour and whole blood samples for molecular analysis can be challenging, emphasising the need for innovative molecular and immune labelling methods for formalin-fixed paraffin-embedded samples.

In clinical practice, there is a notable difference in the choice of treatment regimen between NET G3 and NECs. The standard first-line chemotherapy regimen for NECs typically involves a combination of platinum and etoposide. For NET G3, guidelines typically advise the use of treatment strategies comparable to those recommended for NET G2. However, further large-scale studies are needed to identify the most suitable therapeutic options (11). Therefore, the current study aimed to explore a targeted HG-NEN mutation model in conjunction with lineage alterations at the primary tumour site to identify NECs and NET G3, with the goal of guiding the development of more rational and standardised treatment approaches.

## 2 Materials and methods

### 2.1 Case selection

This study included a total of 38 cases of patients with GEP-HG-NENs who underwent biopsy, puncture, or surgery or who received consultation at Beijing Luhe Hospital Affiliated to Capital Medical University, from January 1, 2004, to May 1, 2022. Three senior pathologists performed the diagnostic evaluation based on the criteria outlined in the 5th edition of the 2019 WHO Classification of Tumours of the Digestive System. Where discrepancies in pathological grading occurred, a consensus was reached through consultation and discussion among the three pathologists. The clinicopathological characteristics of all cases were summarised and analysed.

The need for informed consent was waived owing to the retrospective nature of this study, which only involved an analysis of previously collected data and utilised anonymised data obtained from existing records. Nonetheless, strict confidentiality and adherence to ethical guidelines regarding patient privacy were ensured.

### 2.2 Immunohistochemistry and quantitative real-time polymerase chain reaction

Immunohistochemical staining and analysis were conducted on paraffin-embedded samples from 38 HG-NEN cases. The primary antibodies used in this study were chromogranin A (CHGA), synaptophysin (SYP), CD56, death domain associated protein (DAXX), ATRX chromatin remodeler (ATRX), TP53, RB, insulin-related protein 1 (INSM1), SSTR2, recombinant human CLU, and MKI67. The cell membrane or cytoplasmic markers were positively stained for SYP, CHGA, CD56, and CLU, whereas nuclear staining was observed for DAXX, ATRX, TP53, RB, INSM1, and MKI67. The SSTR2 expression was interpretated and scored using the following scale, based on the HER2 criteria for gastric cancer, as recommended by the Chinese consensus on the pathological diagnosis of gastrointestinal and pancreatic neuroendocrine neoplasms in 2020 (17): 0, no discoloration; 1+, weak membrane staining in at least 10% of tumour cells; 2+, weak to moderately intense membrane staining in at least 10% of tumour cells; and 3+, strong membrane staining in at least 10% of tumour cells. Because *TP53* expression in GEP-HG-NENs lacks a standardised evaluation method, in this study, mutations were identified based on moderate–intensity positivity (>30%) or no staining (18, 19). The scoring criteria for the remaining immunohistochemical markers were as follows: >10%, negative; 10% to 25%, 1+; 25% to 50%, 2+; <50%, 3+. *KRAS* and *BRAF V600E* mutations were screened in 34 cases of GEP-HG-NENs, including 1 and 3 cases of NET G3 and SCNEC 4, respectively that predated 2012 and were excluded because of specimen size and storage period considerations.

### 2.3 Statistical analysis

Data were analysed using descriptive statistics, and differences in frequencies were assessed using the chi-squared (χ^2^) test or Fisher’s exact test. Logistic regression was used to assess categorical variables in both the multivariate and univariate analyses. Overall survival was assessed from the date of diagnosis to the date of death or last follow-up. Survival curves were drawn using the Kaplan–Meier method, and differences between groups were assessed using the log-rank test. Univariate and multivariate Cox proportional hazards regression analyses were conducted to assess the prognostic significance of various clinical and histopathological characteristics. Data analysis was performed using SPSS software version 26.0 (IBM Corp., Armonk, NY, USA). All tests were two-sided, and statistical significance was defined as *p* < 0.05.

## 3 Results

### 3.1 Clinicopathological features

**Figure 1:**
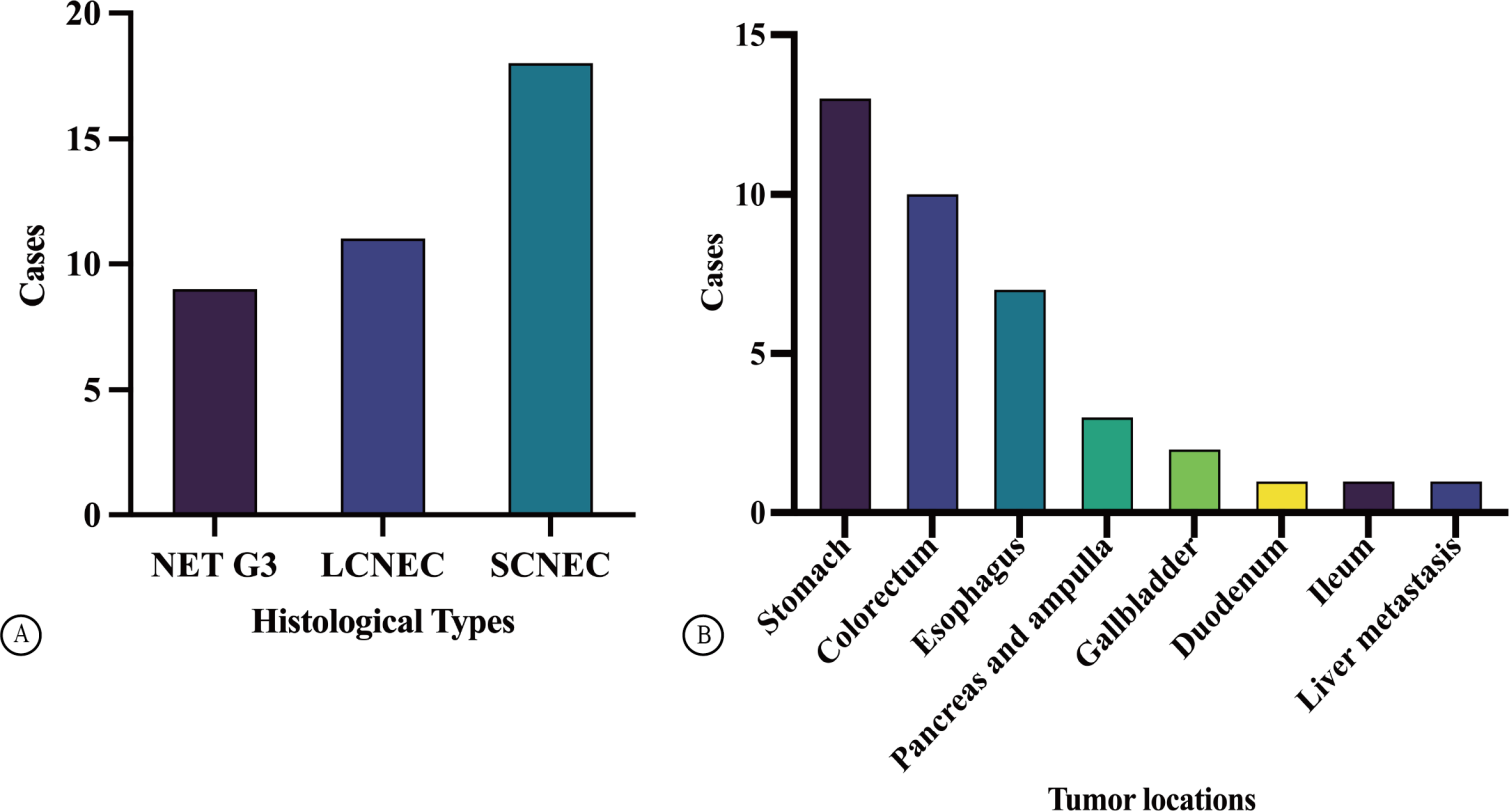
Case distribution of GEP HG NENs **(A)**. The distribution of tumor locations in GEP HG NENs **(B)**.

A total of 181 patients with GEP-NENs were treated at our hospital from January 1, 2004, to May 1, 2022. Overall, 38 cases (21.0%) were confirmed to be HG-NENs. Among these, 9 and 29 cases were diagnosed as NET G3 (23.7%) and NECs (76.3%), respectively, including 11 and 18 LCNEC (37.9% of NECs) and SCNEC (62.1% of NECs) cases, respectively (**Figure 1A**). Of the 38 patients with HG-NENs, 22 (57.9%) and 16 (42.1%) were male and female (age range: 49–86 years, average age: 66 years). Specifically, nine NET G3 cases involved seven male and two female patients, with an average age of 72.5 years.

Among 29 cases of NECs, 11 were LCNEC cases (involving five and six male and female patients, respectively; median age: 69 years) with a median age of 69 years, whereas 18 were SCNEC cases (involving nine male and female patients each; average age: 67.5 years). The specimen types collected were needle biopsy and surgical resection specimens in 31 cases (81.6%) and specimens in 7 cases (18.4%). Tumours originated in different locations—specifically, in the stomach, colorectum, oesophagus, pancreas and ampulla, and gallbladder in 13 (34.2%), 10 (26.3%), 7 (18.4%), 3 (7.9%), and 2 (5.2%) cases, respectively, and in the duodenum, ileum, and liver in 1 case each (2.6% each, **Figure 1B**). There was a higher proportion of male than female patients with HG-NENs (male-to-female ratio, 1.4:1); among the 9 and 29 cases of NET G3 and NECS, the male-to-female ratio was 3.5:1 and 0.9:1, respectively. Most tumours were located in the stomach (13/38), followed by the colorectum and oesophagus. Interestingly, four cases (44.4%) of stomach-originating HG-NENs were classified as NET G3.

Nerve invasion was observed in three cases (42.9%) among all those involving patients who had surgically resected specimens, which consisted of one and two cases of LCNEC and SCNEC, respectively. In addition, intravascular tumour thrombus and SCNEC were observed in three (42.9%) and five (71.4%), cases, respectively, whereas surrounding lymph node metastasis occurred in two LCNEC cases and three SCNEC cases. The most common distant metastatic sites were the liver, lungs, and peritoneum during the follow-up period (**Table 1**). No significant correlation (*p >* 0.05) was found between the patients’ sex, age, smoking history, drinking history, MKI67 index, and histological type.

**Table 1:** Metastasis between different histological types.

Metastatic sites	NET G3 (%)	LCNEC (%)	SCNEC (%)
liver	2 (22.2)	4 (36.4)	6 (33.3)
lungs	1 (11.1)	0 (0)	3 (16.7)
peritoneal metastasis	1 (11.1)	1 (9.1)	0 (0)

### 3.2 Pathological features

**Figure 2:**
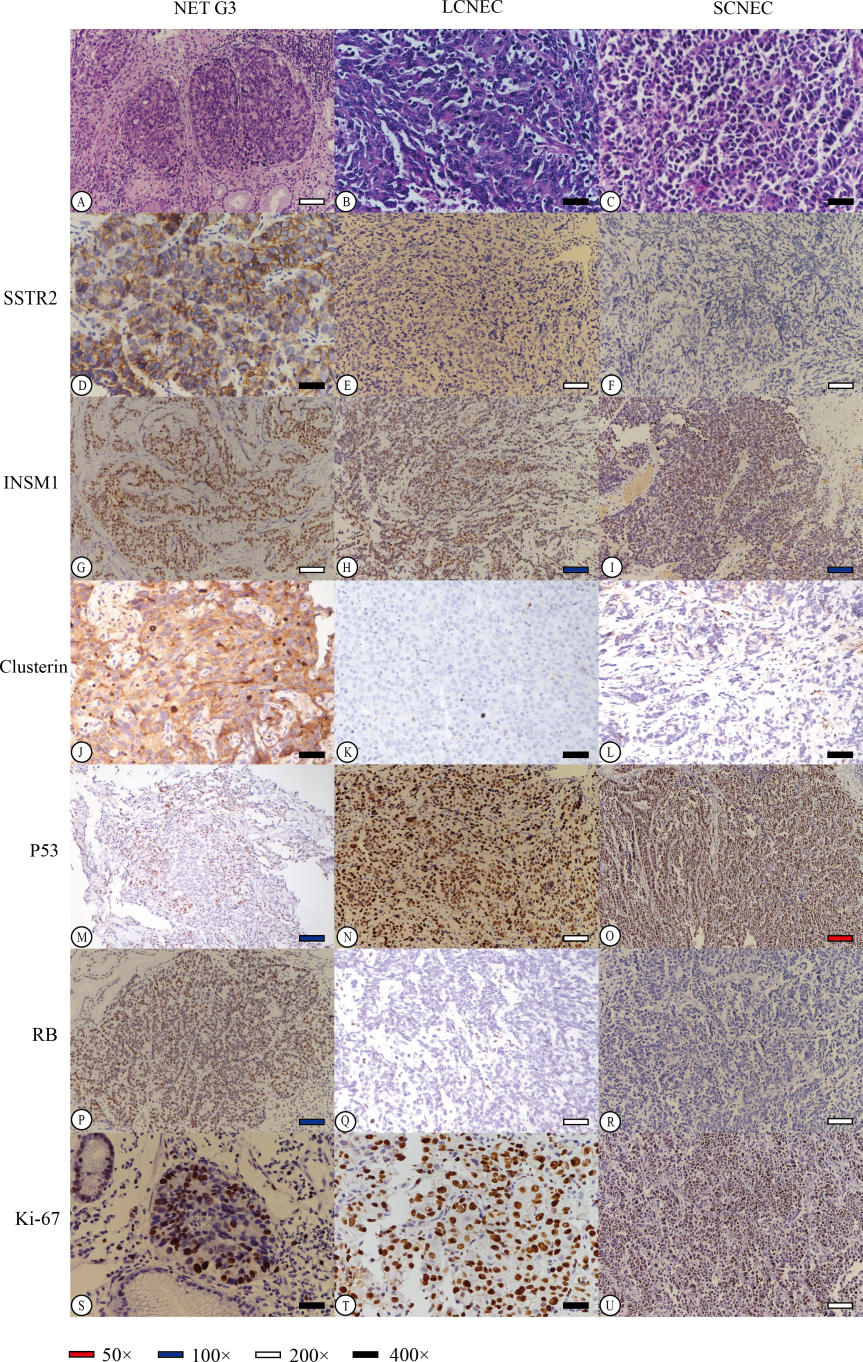
Hematoxylin-Eosin (HE) staining of different neuroendocrine tumor subtypes revealed distinct characteristics. NET G3 **(A)** displayed well-differentiated tumor cells with uniform morphology and slightly eosinophilic cytoplasm. LCNEC **(B)** exhibited fine cytoplasm and prominent hyperchromatic large nucleoli, while SCNEC **(C)** showed sparse cytoplasm, inconspicuous cell nucleus, and active caryomitosis. We carefully selected representative stained regions from various histological subtypes to effectively demonstrate the staining patterns of each immunohistochemical marker (D-U). Diffuse strong membrane staining of SSTR2 was observed in NET G3, with a score of 4 **(D)**. Conversely, in LCNEC and SCNEC, tumor cells showed no expression of SSTR2, resulting in a score of 0 **(E, F)**. Furthermore, all tumor cells tested positive for INSM1 in NET G3, LCNEC, or SCNEC **(G–I)**. Clusterin staining in tumor cells of NET G3 exhibited strong positive staining in both the cell membrane and cytoplasm, with a score of 3+ **(J)**. However, LCNEC and SCNEC showed negative expression of clusterin in tumor cells, with a score of 0 **(K, L)**. Additionally, P53 expression in tumor cells of NET G3 was wild-type **(M)**, while LCNEC and SCNEC displayed mutant p53 expression (missense mutation) **(N, O)**. RB protein expression was positive in NET G3, scoring 3+ **(P)**, but was lost in tumor cells of LCNEC and SCNEC, scoring 0 **(Q, R)**. The Ki-67 index in tumor cells was approximately 35% in NET G3 **(S)**, 85% in LCNEC **(T)**, and 90% in SCNEC **(U)**.

NET G3 appeared uniform in size with a round or oval shape and exhibited mild-to-moderate atypia. The cytoplasm appeared fine or finely granular, basophilic or dichromatic, whereas the nuclear chromatin was coarse and resembled pepper–salt-like granules. Mitosis was rare, and focal necrosis resembled a comedo (**Figure 2A**). In contrast, the NECs demonstrated diffuse lamellar growth, irregular tumour cell arrangement, lack of organ structure, significant cellular atypia, prominent mitotic activity, and extensive areas of necrosis. LCNEC had a relatively abundant cytoplasm, obvious nuclear atypia, and large prominent nucleoli (**Figure 2B**). In contrast, SCNEC exhibited sparse cytoplasm, high nucleus-to-cytoplasm ratio, and hyperchromatic naked nuclei with inconspicuous nucleoli (**Figure 2C**).

### 3.3 Analysis of immunohistochemical results

The immunohistochemical analysis indicated that 47.4% of HG-NENs expressed SSTR2 at a level above 1+ based on the HER2 criteria for gastric cancer (**Table 2**). Furthermore, eight of the nine NET G3 cases expressed SSTR2, mainly at scores of 2+ and 3+. Additionally, weakly positive SSTR2 expression was detected in four and six LCNEC and SCNEC cases, respectively, in areas with better histological differentiation than others. Moreover, a statistically significant difference in SSTR2 positivity was observed between NECs and NET G3 (*p =* 0.012) (**Figures 2D–F**).

**Table 2:** Correlation analysis between various immunohistochemical indexes and histological types.

	NET G3(%)	LCNEC(%)	SCNEC(%)	P value	NET G3(%)	NEC(%)	P value
				(<0.05)			(<0.05)
SSTR2							
0	1 (11.1)	7 (63.6)	12 (66.7)	0.090	1 (11.1)	19 (65.5)	0.012^*^
1+	2 (22.2)	1 (9.1)	3 (16.7)		2 (22.2)	4 (13.8)	
2+	3 (33.3)	2 (18.2)	2 (11.1)		3 (33.3)	4 (13.8)	
3+	3 (33.3)	1 (9.1)	1 (5.6)		3 (33.3)	2 (6.9)	
Rb							
0	1 (11.1)	3 (27.4)	12 (66.7)	0.001^*^	1 (11.1)	15 (51.7)	0.110
1+	3 (33.3)	0 (0)	5 (27.8)		3 (33.3)	5 (17.2)	
2+	4 (44.4)	6 (54.4)	1 (5.6)		4 (44.4)	7 (24.1)	
3+	1 (11.1)	2 (18.2)	0 (0)		1 (11.1)	2 (6.9)	
P53							
Wild-type form	5 (55.6)	1 (9.1)	4 (22.2)	0.076	5 (55.6)	5 (17.2)	0.036^*^
Mutation	4 (44.4)	10 (90.9)	14 (77.8)		4 (44.4)	24 (82.8)	
Ki-67 index							
<55	8 (88.9)	2 (18.2)	1 (5.6)	0.000026^*^	7 (87.5)	3 (14.3.2)	0.000028^*^
≥55	1 (11.1)	9 (81.8)	17 (94.4)		1 (12.5)	18 (85.7)	
ATRX							
0	1 (11.1)	2 (18.2)	3 (16.7)	0.484	1 (11.1)	5 (17.2)	0.219
1+	3 (33.3)	1 (9.1)	2 (11.1)		3 (33.3)	3 (10.3)	
2+	3 (33.3)	1 (9.1)	5 (27.8)		3 (33.3)	6 (20.7)	
3+	2 (22.2)	7 (63.6)	8 (44.4)		2 (22.2)	15 (51.7)	
Clusterin							
0	0 (0)	7 (63.6)	12 (66.7)	0.001^*^	0 (0)	19 (65.5)	0.0001^*^
1+	1 (11.2)	2 (18.2)	2 (11.1)		1 (11.2)	4 (13.8)	
2+	4 (44.4)	1 (9.1)	4 (22.2)		4 (44.4)	5 (17.2)	
3+	4 (44.4)	1 (9.1)	0 (0)		4 (44.4)	1 (3.4)	
DAXX							
0	5 (55.6)	4 (36.4)	6 (33.3)	0.918	5 (55.6)	10 (34.5)	0.634
1+	3 (33.3)	4 (36.4)	5 (27.8)		3 (33.3)	9 (31.1)	
2+	1 (11.1)	2 (18.2)	5 (27.8)		1 (11.1)	7 (24.1)	
3+	0 (0)	1 (9.1)	2 (11.1)		0 (0)	3 (10.3)	

*: *p* < 0.05, indicating a statistically significant difference.

We found that the total positivity rate for *INSM1* expression was 86.8% (33/38), with 77.8% (7/9) and 89.7% (26/29) in the NET G3 and NEC groups, respectively (**Figures 2G–I**). CLU was expressed in 50.0% (19/38) of the cases, with 100% and 34.5% positivity in the NET G3 and NEC groups, respectively (**Figures 2J–L**). *TP53* mutation was present in 81.6% (31/38) of cases, with mutation rates of 44.4% (4/9) and 82.8% (24/29) in the NET G3 and NEC groups, respectively (**Figures 2M–O**). RB expression was loss in 11.1% (1/9) and 55.2% (16/29) of the cells in the NET G3 and NEC groups, respectively (**Figures 2P–R**). Specifically, the expression rates of SYP, CHGA, and CD56 were 92.1%, 57.9%, and 60.5%, respectively.

The MKI67 proliferative index varied greatly between the NET G3 and NECs groups, with a median of approximately 40% and 70% and range of 25%–70% and 50%–90%, respectively. The correlation analysis showed that an MKI67 index between 20% and 55% was more likely to indicate NET G3. A 55% cutoff value was used for the MKI67 index; 11 cases fell between 20% and 55%, whereas 27 cases were at ≥55%, including 26 cases of NECs (**Figures 2S–U**). *DAXX* and *ATRX* gene expression was positive in 60.5% (23/38) and 84.2% (32/38) of the cases, respectively. SSTR2, RB, TP53, CLU, and *MKI67* expression levels were significantly different between the NET G3 and NEC groups of HG-NENs (*p <* 0.05, **Table 2**), while other indicators did not show statistically significant differences.

### 3.4 qRT-PCR of *KRAS* and *BRAF V600E*

**Figure 3:**
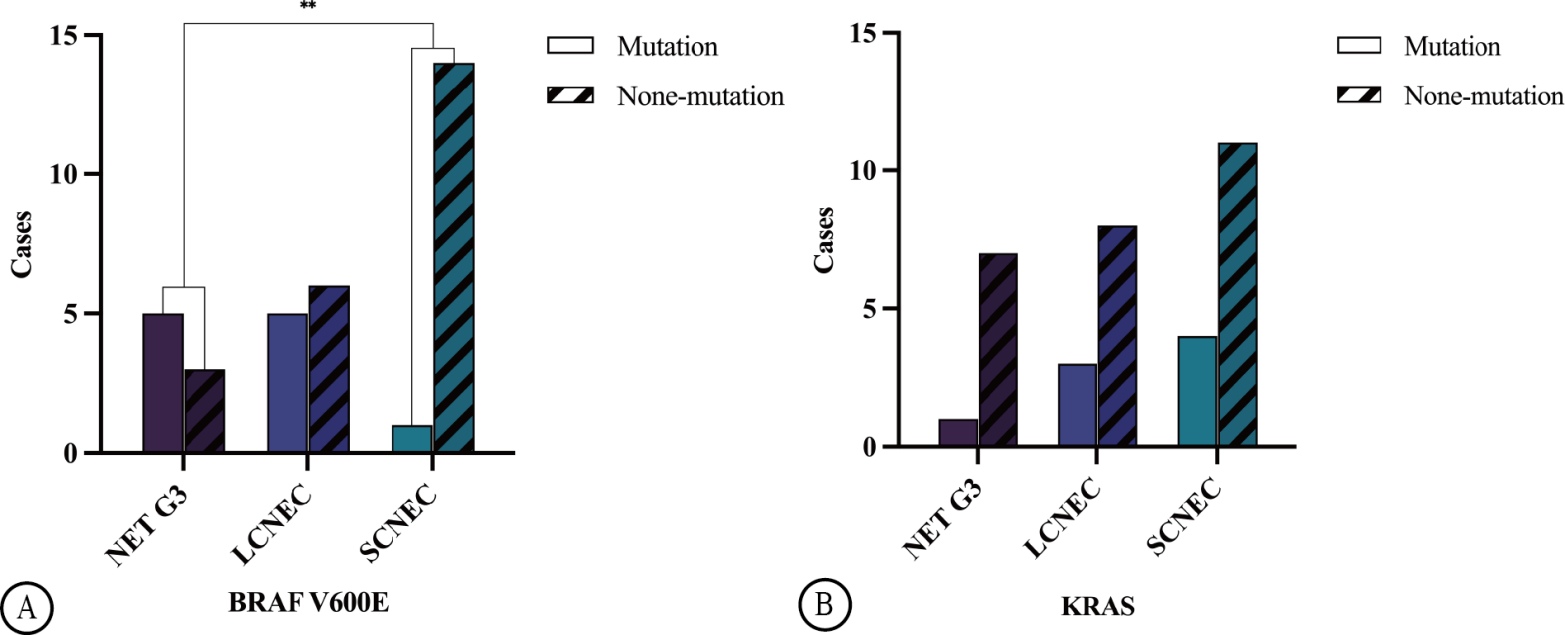
Illustrates the distribution of BRAF V600E mutation in GEP HG-NENs. A statistically significant disparity (**p=0.0086) was observed between NET G3 and SCNEC **(A)**. The distribution of KRAS mutation in GEP HG-NENs is also shown in the figure **(B)**.

Among the 34 HG-NEN cases, 11 (32.4%) harboured the *BRAF V600E* mutation. This included five cases of NET G3 (62.5%) and six cases of NECs (23.1%), which consisted of one and five cases of SCNEC (6.7%) and LCNEC (45.5%), respectively. A significant difference in *BRAF V600E* mutation was observed between NET G3 and SCNEC (*p =* 0.0086) (**Figure 3A**). Mutation rates varied across primary sites: 60.0% in the colon (3/5), 20.0% in the rectum (1/5), 41.7% in the stomach (5/12), and 33.3% in the oesophagus (2/6). *KRAS* mutations were observed in 8 of the 34 HG-NEN cases (23.5%). This included one case of NET G3 (12.5%) and seven of NECs (26.9%), which comprised three and four cases of LCNEC (27.3%) and SCNEC (26.7%), respectively (**Figure 3B**).

There was no significant difference in *KRAS* mutation frequency between the different histological types (*p =* 0.763), while the mutation rate varied across primary sites, with 40.0% and 25% in the colorectum (4/10) and stomach (3/12), respectively. No *KRAS* mutations were detected in the primary oesophageal cancer cases. Loss of RB protein expression was identified in 16 cases, with one case involving NET G3 while the remaining 15 involved NECs and consisted of 3 and 12 cases of LCNEC and SCNEC, respectively. Statistically significant differences were observed in RB protein expression among the NET G3, LCNEC, and SCNEC groups (*p =* 0.001). Further comparison revealed that SCNEC showed higher susceptibility to the loss of RB protein expression than NET G3 and LCNEC did.

### 3.5 Prognostic analysis

Complete follow-up data was obtained for 35 of the 38 patients with HG-NENs, whereas two and one patient with NECs and NET G3, respectively, were lost to follow-up. The median follow-up time was 12 (range: 3–86) months. The median survival time (MST) for the NET G3 and NEC groups was 25 (range: 5–80, average: 32) and 11 (range: 3–86, average 16) months, respectively. There were 23 deaths (60.5%), consisting of 7, 11, and 5 in the LCNEC (63.6%), SCNEC (61.1%), and NET G3 (55.6%) groups, respectively. Organ failure due to liver and lung metastases or multiple systemic metastases or a combination of these was the primary cause of death (**Table 1**).

**Figure 4:**
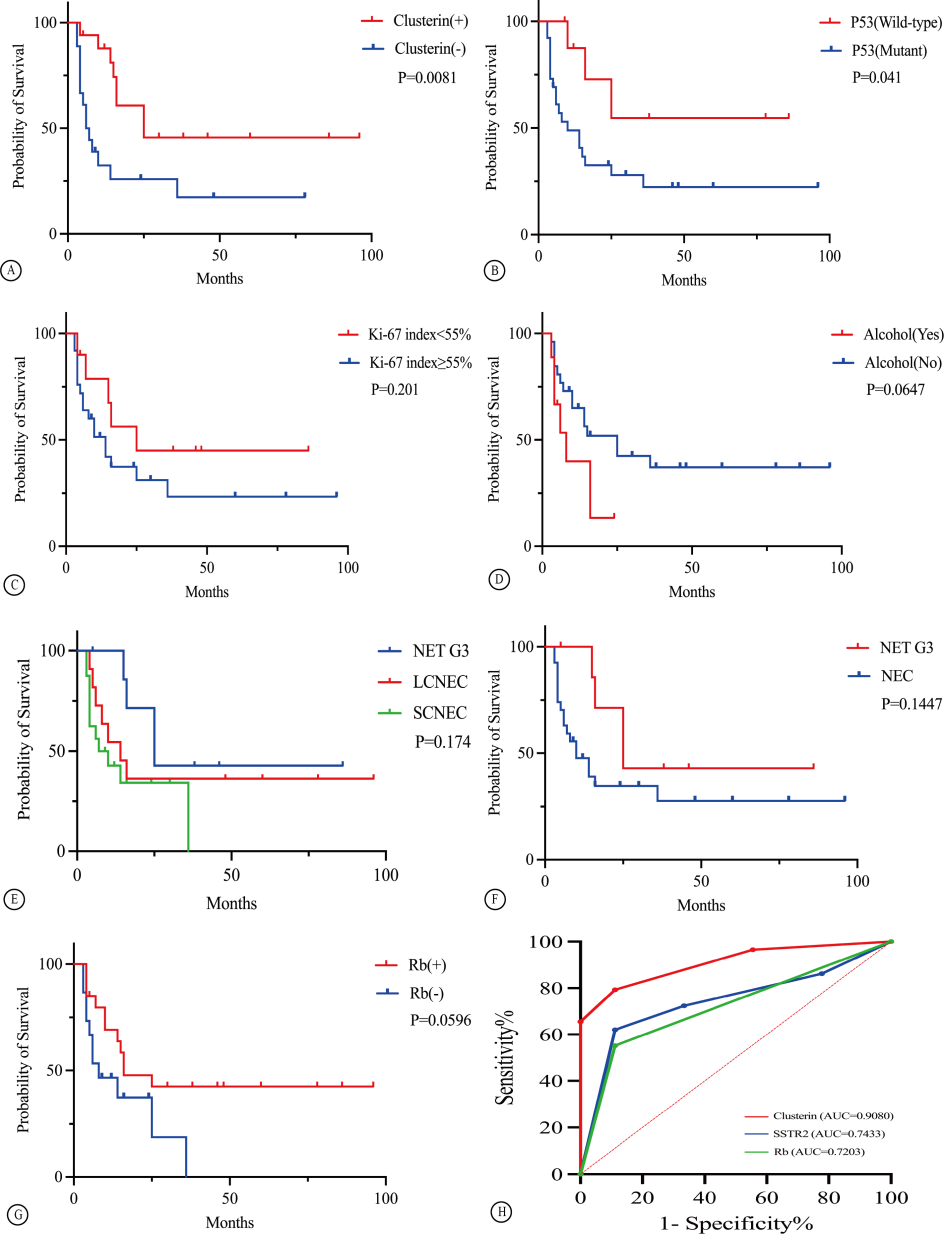
Overall survival of patients with GEP HG-NENs of various clinical and histopathological characteristics **(A–G)**. The ROC curves for the Rb, Clusterin, and SSTR2 markers **(H)**.

**Figure 5:**
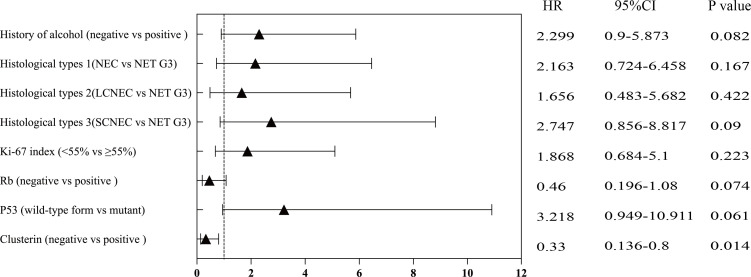
Univariate analysis (based on selected variables) of overall survival in various subgroups.

The Kaplan–Meier survival curve for CLU (p = 0.0081, **Figure 4A**) and TP53 (p = 0.041, **Figure 4B**) indicated statistically significant differences between the two different groups. Univariate and multivariate Cox regression analyses showed that the expression of the immunohistochemical index CLU (p = 0.014, **Figure 5** and **Table 3**) significantly affected the patients’ survival time and outcomes.

**Table 3:** Univariate and multivariable analysis (based on selected variables) of overall survival.

	HR	95%CI	*p*-value
Univariate analysis			
History of alcohol (negative vs positive )	2.299	0.9-5.873	0.082
Histological types 1(NEC vs NET G3)	2.163	0.724-6.458	0.167
Histological types 2(LCNEC vs NET G3)	1.656	0.483-5.682	0.422
Histological types 3(SCNEC vs NET G3)	2.747	0.856-8.817	0.09
Ki-67 index (<55% vs ≥55%)	1.868	0.684-5.1	0.223
Rb (negative vs positive )	0.46	0.196-1.08	0.074
P53 (wild-type form vs mutant)	3.218	0.949-10.911	0.061
Clusterin (negative vs positive )	0.33	0.136-0.8	0.014^*^
Multivariate analysis			
Clusterin (negative vs positive )	0.33	0.136-0.8	0.014^*^

*: *p* < 0.05, indicating a statistically significant difference.

In this study, an MKI67 proliferation index of 55% was used as the cutoff for analysing different factors. The survival analysis did not show a statistically significant difference (*p =* 0.201, **Figure 4C**). Other factors such as sex, age, and history of smoking and drinking did not show a statistically significant correlation with survival time (all *p >* 0.05; **Figures 4D–G**). The receiver operating characteristic (ROC) curve analysis indicated that the positive expression of the immunohistochemical markers CLU, SSTR2, and RB has significant implications for diagnosing NET G3 (**Figure 4H**).

## 4 Discussion

In this study we investigated various immunohistochemical and molecular biological markers and their association with clinicopathological features in GEP-HG-NENs. The fifth edition of the WHO Classification of Tumours of the Digestive System places greater focus on precisely categorising NET G3 and NECs according to the varying levels of differentiation seen in pathological tissue morphology. However, this approach may result in discrepancies among pathologists. Grossly, NET G3 tumours typically present as a solid nodule with a raised or polyp-like appearance, while some show ulceration, medium-hard texture, and greyish-white or greyish-yellow surface. It exhibits outwardly expanding growth with a distinct demarcation.

In contrast, NECs often display an irregular ulcer or cauliflower-like bulge, are frequently accompanied by haemorrhage or necrosis, and have a brittle texture. When these tumours occur in the colorectum, they tend to grow around the circumference, leading to luminal narrowing. Some cystic lesions may have poorly defined borders with the surrounding tissues. Microscopically, NET G3 resembles a well-differentiated NENs shows organ-like structures or growth in trabecular, ribbon-, and gland-like patterns. Certain cells form pseudo-rosettes. In our study, the slides were reviewed by a minimum of three senior pathologists and reclassified according to the most recent classification criteria.

We found a higher prevalence of HG-NENs in male patients than in female patients, with most tumours being located in the stomach, followed by the colorectum and oesophagus. Interestingly, the classification of all four stomach-originating HG-NEN cases as NET G3 differed slightly from those previously reported, suggesting that NET G3 primarily occurs in the pancreas (11). This discrepancy may be attributed to the adoption of new classification standards and the variations in specimen types across different research institutions. Busico et al. (2) further classified HG-NENs into three subgroups (namely, NET G3, NECs <55%, and NECs ≥55%) and reported notable variations in survival rates across these subgroups. However, in our study, the Cox regression analysis indicated that patient survival duration was not significantly correlated with an MKI67 proliferative index of 55%.

In this study, we also found that GEP-HG-NENs were positive for immunohistochemical markers with varying degrees of diagnostic significance. The histological types were further classified into NET G3, LCNEC, and SCNEC, with distinct differences observed in immunohistochemical indicators such as SSTR2, TP53, RB, and CLU. The SSTR2 expression in gastric cancer was interpreted based on the HER2 interpretation criteria outlined in the 2020 Chinese consensus on the pathological diagnosis of gastrointestinal and pancreatic neuroendocrine neoplasms. In our previous study, Fisher’s test revealed a statistically significant difference among NET G3, LCNEC, and SCNEC when SSTR2 expression was negative; however, after including nine new cases, no significant difference was observed among the three groups (20).

In this study, the RB, ATRX, DAXX, and CLU levels were interpreted based on the SSTR2 interpretation standard. Previous studies reported high CLU expression in LG-NENs (10), which is similar to our present finding in which the NET G3 group exhibited a significantly higher CLU expression and ≥2+ frequency rate than the NEC group did. Notably, tumours originating from the midgut were not included in the cases studied, suggesting that the CLU expression level was potentially lower in NET G3 from the midgut than in those originating elsewhere (21). Strong CLU expression has been hypothesised to be probably more indicative of NET G3 in cases where cell morphology shows a deceptive divergence.

Cox regression analysis indicated that patients with positive CLU had a 0.33-fold lower risk of mortality than those with negative expression do. Consequently, the use of the immunohistochemical marker CLU as a prognostic indicator of survival in patients with GEP-HG-NENs has significant clinical implications. Furthermore, the potential use of the CLU inhibitor custirsen (OGX-111) in GEP-HG-NENs has been proposed to improve the survival prognosis of GEP-HG-NENs (22–24). We found that the prevalence of *TP53* mutation was significantly higher in NECs than it was in NET G3. However, there were no significant difference in P53 mutation between LCNEC and SCNEC.

Among the four cases of *TP53* mutation in NET G3, two were located in the oesophagus and one each in the colorectum and gastric body. These patients showed metastases to the liver or lungs during the later stages of disease progression. This suggests that the *TP53* mutation may be associated with the aggressive behaviour of HG-NENs, which aligns with previous findings (25). Previous studies have proposed that combined immunohistochemical staining of SSTR2, TP53, and RB could facilitate in distinguishing HG-NENs that are morphologically challenging to differentiate (8).

Theoretically, high and robust SSTR2 expression in combination with wild-type TP53 and RB protein expression, is more indicative of NET G3 than it is of NECs, whereas the opposite expression pattern tends to indicate NECs. Our study revealed a high level of SSTR2 expression in the NET G3 group and a higher prevalence of *TP53* missense mutation in the NEC group than in the NET G3, which is consistent with previous research findings (19). Nevertheless, a substantial amount of clinical sample data would be necessary to determine the correlation between these target immunohistochemical markers and their prognostic significance in GEP-HG-NENs.

Recent studies have reported that approximately 20.8% and 9.9% of patients with colorectal NECs and GEP-NENs, respectively, harbour the *BRAF V600E* mutation (26, 27). Targeting the *BRAF* oncogene may serve as a promising therapeutic approach for individuals with *BRAF*-mutated colorectal NECs. In our study, the *BRAF V600E* mutation was identified in 32.4% of the HG-NEN cases. Interestingly, the colon exhibited the highest BRAF mutation rate among the various primary tumour sites, which is consistent with previous research findings (26).

Significant differences were observed between NET G3 and SCNEC in our study, and the mutation rate of NET G3 was higher than that of NECs, which contradicts the existing literature reports (27). This discrepancy could be due to the small sample size and variations in the qRT-PCR detection results from the different primary tumour sites. Therefore, further experimental validation using a larger sample size is warranted. The implications of *BRAF* mutation detection vary across different histological types and primary tumour sites, which highlights the need for a comprehensive investigation with a larger sample size to clarify the correlation between these factors.

A recent study found *KRAS* mutations in 31% and 25% of NECs from the colon and rectum, respectively, and identified *KRAS* mutations in three cases of primary gastric NECs but not in NET G3 (26). In our study of 34 HG-NEN cases, although *KRAS* gene mutations were identified NET G3 and NEC cases, no significant differences were observed among different histological types. The rate of *KRAS* mutations in the colorectum was notably higher than that in the stomach and other sites, which is consistent with previous findings (28).

Among the eight cases of NET G3, *KRAS* mutation was detected in one, which involved a 61-year-old male patient with a history of smoking and drinking. The patient presented with upper gastrointestinal bleeding and elevated blood alpha fetoprotein (AFP) and CEA cell adhesion molecule (CEA) levels and survived for only 16 months. These findings may be useful in developing clinical treatment strategies, suggesting that a *KRAS* mutation in NET G3 may lead to poor survival outcomes and rapid disease progression. Detecting *KRAS* mutations is essential for guiding treatment, but further analyses of larger sample sizes are needed to confirm its correlation with specific types of GEP-HG-NENs.

Nevertheless, the current study has certain limitations. Owing to the rarity of GEP-HG-NENs, the number of patients included in this study was relatively small, and the retrospective study spanned 18 years. Although statistical errors were minimised and patient survival was assessed using Fisher’s exact test and the Kaplan–Meier method, which are suitable for a small sample size, these factors may still limit the statistical power and generalisability to larger populations. The extended time frame of the collected cases introduced some heterogeneity within the patient cohort, making it more challenging to draw definitive conclusions. This heterogeneity included variations in the tumour type, clinical staging, and treatment approaches. Additionally, further validation of *KRAS* and *BRAF V600E* mutations in these tumours requires larger-scale sequencing data. In future research, we plan to collect more clinical samples to arrive at more precise conclusions.

## 5 Conclusion

The recent update on the GEP-HG-NEN standard highlights significant differences in pathogenesis, pathological diagnosis, clinical treatment, and prognosis between NET G3 and NECs. Therefore, clinical pathologists essentially must have a comprehensive understanding of the histological and cytological morphological characteristics of tumours to accurately classify HG-NENs. The use of valuable immunohistochemical and molecular pathological indicators in combination with the MKI67 proliferation index is essential for comprehensive analysis when indicated. Our research findings can aid clinical pathologists in distinguishing NET G3 from NECs; however, there are still some limitations. We will persist in gathering relevant cases to broaden the sample size and achieve more dependable results. Enhancing the comprehension of GEP-HG-NENs will facilitate not only the development of standardised and rational treatment plans in clinical practice but also the implementation of appropriate prognostic management.

## Data Availability

The original contributions presented in the study are included in the article/supplementary material. Further inquiries can be directed to the corresponding author.
